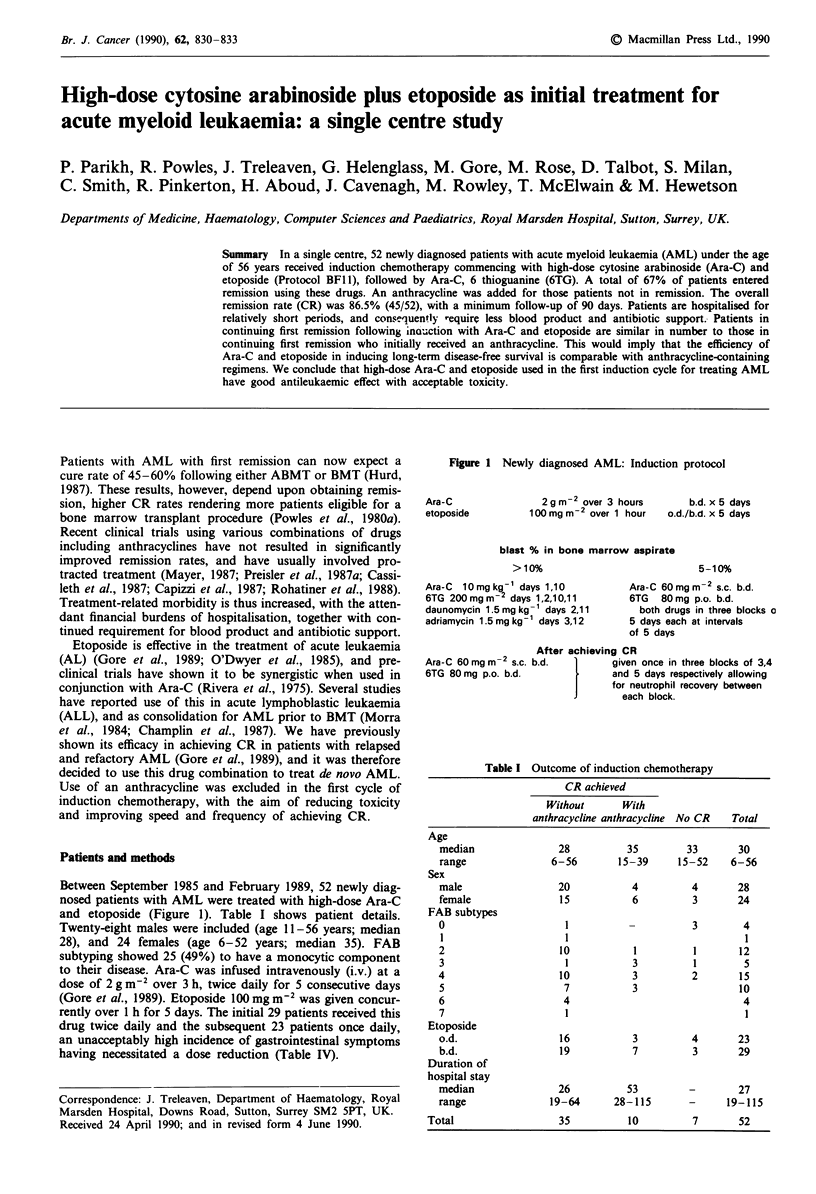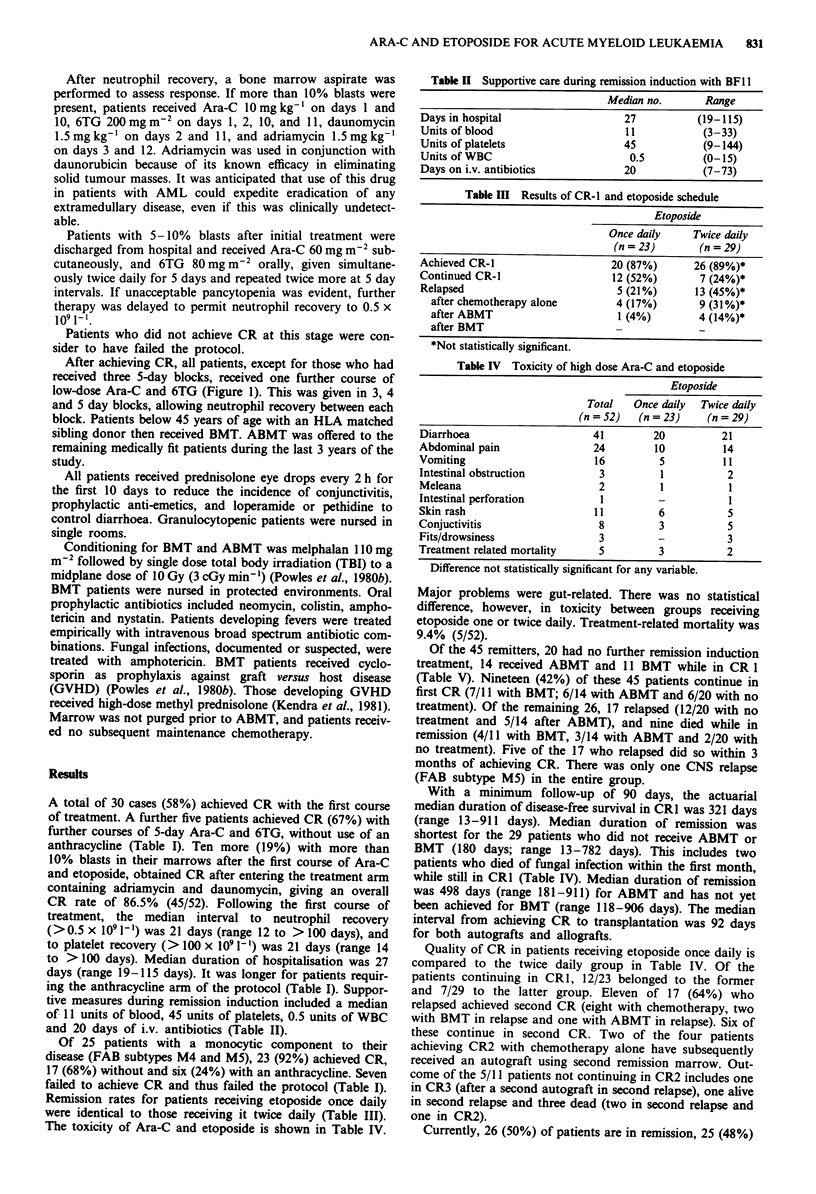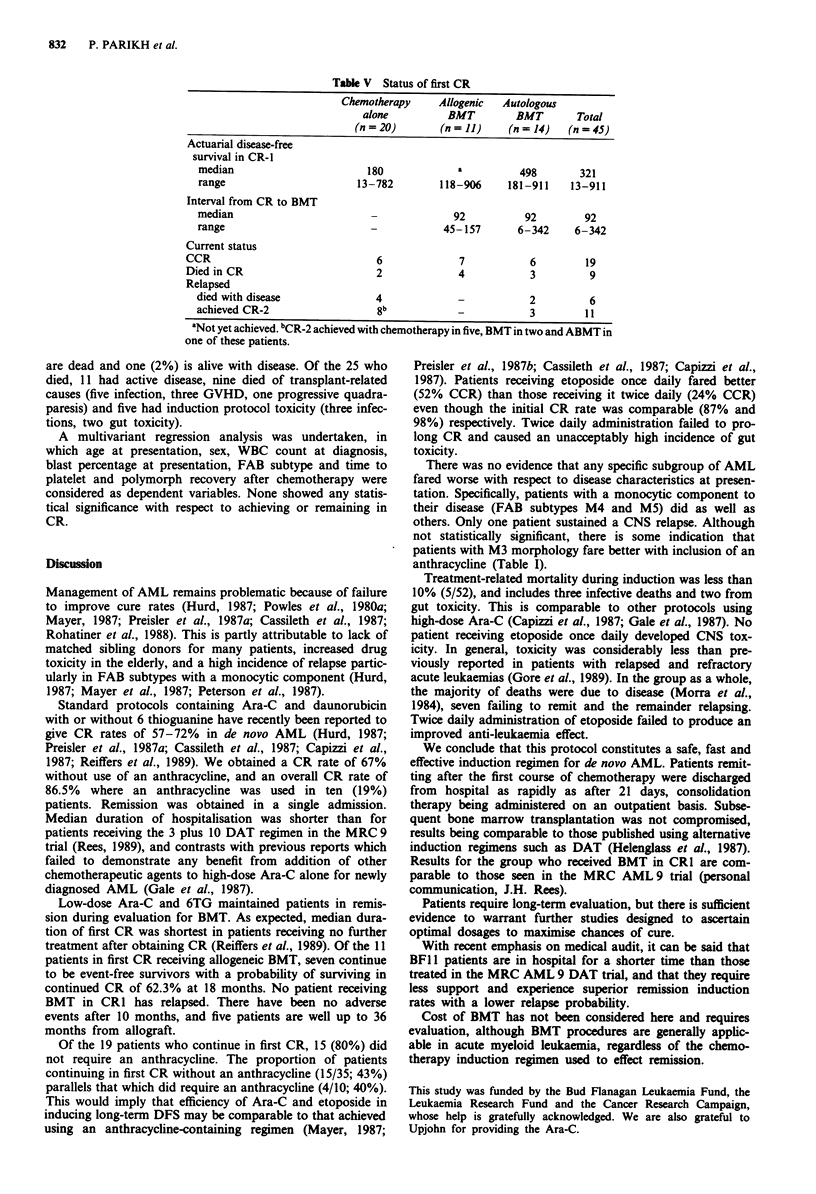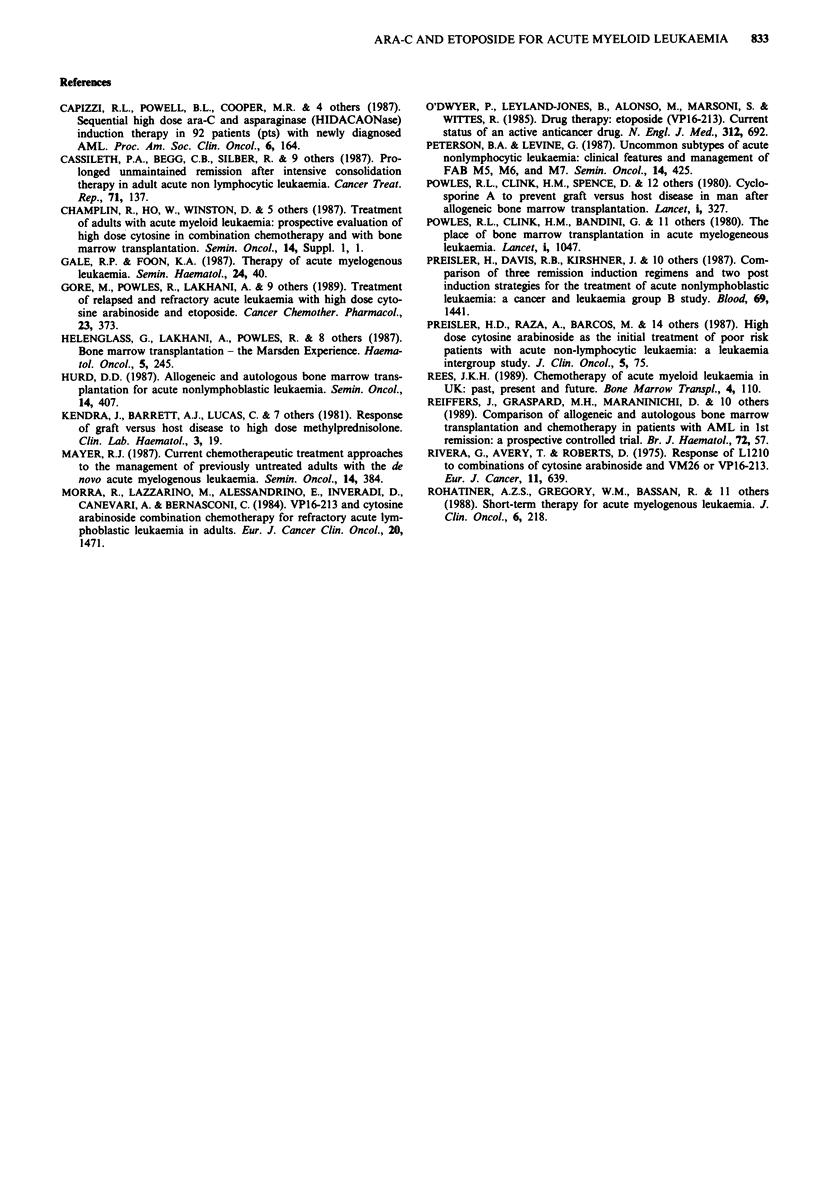# High-dose cytosine arabinoside plus etoposide as initial treatment for acute myeloid leukaemia: a single centre study.

**DOI:** 10.1038/bjc.1990.387

**Published:** 1990-11

**Authors:** P. Parikh, R. Powles, J. Treleaven, G. Helenglass, M. Gore, M. Rose, D. Talbot, S. Milan, C. Smith, R. Pinkerton

**Affiliations:** Department of Medicine, Royal Marsden Hospital, Sutton, Surrey, UK.

## Abstract

In a single centre, 52 newly diagnosed patients with acute myeloid leukemia (AML) under the age of 56 years received induction chemotherapy commencing with high-dose cytosine arabinoside (Ara-C) and etoposide (Protocol BF11), followed by Ara-C, 6 thioguanine (6TG). A total of 67% of patients entered remission using these drugs. An anthracycline was added for those patients not in remission. The overall remission rate (CR) was 86.5% (45/52), with a minimum follow-up of 90 days. Patients are hospitalised for relatively short periods, and consequently require less blood product and antibiotic support. Patients in continuing first remission following induction with Ara-C and etoposide are similar in number to those in continuing first remission who initially received an anthracycline. This would imply that the efficiency of Ara-C and etoposide in inducing long-term disease-term survival is comparable with anthracycline-containing regimens. We conclude that high-dose Ara-C and etoposide used in the first induction cycle for treating AML have good antileukaemic effect with acceptable toxicity.


Br. J. Cancer (1990), 62, 830-833 © Macmillan Press Ltd., 1990

### High-dose cytosine arabinoside plus etoposide as initial treatment for acute myeloid leukaemia: a single centre study

P. Parikh, R. Powles, J. Treleaven, G. Helenglass, M. Gore, M. Rose, D. Talbot, S. Milan, C. Smith, R. Pinkerton, H. Aboud, J. Cavenagh, M. Rowley, T. McElwain & M. Hewetson

*Departments of Medicine, Haematology, Computer Sciences and Paediatrics, Royal Marsden Hospital, Sutton, Surrey, UK.*

**Summary** In a single centre, 52 newly diagnosed patients with acute myeloid leukaemia (AML) under the age of 56 years received induction chemotherapy commencing with high-dose cytosine arabinoside (Ara-C) and etoposide (Protocol BF11), followed by Ara-C, 6 thioguanine (6TG). A total of 67% of patients entered remission using these drugs. An anthracycline was added for those patients not in remission. The overall remission rate (CR) was 86.5% (45/52), with a minimum follow-up of 90 days. Patients are hospitalised for relatively short periods, and consequently require less blood product and antibiotic support. Patients in continuing first remission following induction with Ara-C and etoposide are similar in number to those in continuing first remission who initially received an anthracycline. This would imply that the efficiency of Ara-C and etoposide in inducing long-term disease-free survival is comparable with anthracycline-containing regimens. We conclude that high-dose Ara-C and etoposide used in the first induction cycle for treating AML have good antileukaemic effect with acceptable toxicity.

Patients with AML with first remission can now expect a cure rate of 45-60% following either ABMT or BMT (Hurd, 1987). These results, however, depend upon obtaining remission, higher CR rates rendering more patients eligible for a bone marrow transplant procedure (Powles *et al.*, 1980*a*). Recent clinical trials using various combinations of drugs including anthracyclines have not resulted in significantly improved remission rates, and have usually involved protracted treatment (Mayer, 1987; Preisler *et al.*, 1987*a*; Cassileth *et al.*, 1987; Capizzi *et al.*, 1987; Rohatiner *et al.*, 1988). Treatment-related morbidity is thus increased, with the attendant financial burdens of hospitalisation, together with continued requirement for blood product and antibiotic support.

Etoposide is effective in the treatment of acute leukaemia (AL) (Gore *et al.*, 1989; O'Dwyer *et al.*, 1985), and preclinical trials have shown it to be synergistic when used in conjunction with Ara-C (Rivera *et al.*, 1975). Several studies have reported use of this in acute lymphoblastic leukaemia (ALL), and as consolidation for AML prior to BMT (Morra *et al.*, 1984; Champlin *et al.*, 1987). We have previously shown its efficacy in achieving CR in patients with relapsed and refactory AML (Gore *et al.*, 1989), and it was therefore decided to use this drug combination to treat *de novo* AML. Use of an anthracycline was excluded in the first cycle of induction chemotherapy, with the aim of reducing toxicity and improving speed and frequency of achieving CR.

### Patients and methods

Between September 1985 and February 1989, 52 newly diagnosed patients with AML were treated with high-dose Ara-C and etoposide (Figure 1). Table I shows patient details. Twenty-eight males were included (age 11-56 years; median 28), and 24 females (age 6-52 years; median 35). FAB subtyping showed 25 (49%) to have a monocytic component to their disease. Ara-C was infused intravenously (i.v.) at a dose of 2 g $m^{-2}$ over 3 h, twice daily for 5 consecutive days (Gore *et al.*, 1989). Etoposide 100 mg $m^{-2}$ was given concurrently over 1 h for 5 days. The initial 29 patients received this drug twice daily and the subsequent 23 patients once daily, an unacceptably high incidence of gastrointestinal symptoms having necessitated a dose reduction (Table IV).

Correspondence: J. Treleaven, Department of Haematology, Royal Marsden Hospital, Downs Road, Sutton, Surrey SM2 5PT, UK.
Received 24 April 1990; and in revised form 4 June 1990.

**Figure 1** Newly diagnosed AML: Induction protocol

| | | |
|---|---|---|
| Ara-C | 2 g $m^{-2}$ over 3 hours | b.d. × 5 days |
| etoposide | 100 mg $m^{-2}$ over 1 hour | o.d./b.d. × 5 days |

**blast % in bone marrow aspirate**

| >10% | 5-10% |
|---|---|
| Ara-C 10 mg $kg^{-1}$ days 1,10<br>6TG 200 mg $m^{-2}$ days 1,2,10,11<br>daunomycin 1.5 mg $kg^{-1}$ days 2,11<br>adriamycin 1.5 mg $kg^{-1}$ days 3,12 | Ara-C 60 mg $m^{-2}$ s.c. b.d.<br>6TG 80 mg p.o. b.d.<br>both drugs in three blocks o<br>5 days each at intervals<br>of 5 days |

**After achieving CR**

| | |
|---|---|
| Ara-C 60 mg $m^{-2}$ s.c. b.d.<br>6TG 80 mg p.o. b.d. | given once in three blocks of 3,4 and 5 days respectively allowing for neutrophil recovery between each block. |

**Table I** Outcome of induction chemotherapy

| | CR achieved | | | |
|---|---|---|---|---|
| | *Without anthracycline* | *With anthracycline* | *No CR* | *Total* |
| Age | | | | |
| median | 28 | 35 | 33 | 30 |
| range | 6-56 | 15-39 | 15-52 | 6-56 |
| Sex | | | | |
| male | 20 | 4 | 4 | 28 |
| female | 15 | 6 | 3 | 24 |
| FAB subtypes | | | | |
| 0 | 1 | - | 3 | 4 |
| 1 | 1 | | | 1 |
| 2 | 10 | 1 | 1 | 12 |
| 3 | 1 | 3 | 1 | 5 |
| 4 | 10 | 3 | 2 | 15 |
| 5 | 7 | 3 | | 10 |
| 6 | 4 | | | 4 |
| 7 | 1 | | | 1 |
| Etoposide | | | | |
| o.d. | 16 | 3 | 4 | 23 |
| b.d. | 19 | 7 | 3 | 29 |
| Duration of hospital stay | | | | |
| median | 26 | 53 | - | 27 |
| range | 19-64 | 28-115 | - | 19-115 |
| Total | 35 | 10 | 7 | 52 |

After neutrophil recovery, a bone marrow aspirate was performed to assess response. If more than 10% blasts were present, patients received Ara-C 10 mg kg$^{-1}$ on days 1 and 10, 6TG 200 mg m$^{-2}$ on days 1, 2, 10, and 11, daunomycin 1.5 mg kg$^{-1}$ on days 2 and 11, and adriamycin 1.5 mg kg$^{-1}$ on days 3 and 12. Adriamycin was used in conjunction with daunorubicin because of its known efficacy in eliminating solid tumour masses. It was anticipated that use of this drug in patients with AML could expedite eradication of any extramedullary disease, even if this was clinically undetectable.

Patients with 5–10% blasts after initial treatment were discharged from hospital and received Ara-C 60 mg m$^{-2}$ subcutaneously, and 6TG 80 mg m$^{-2}$ orally, given simultaneously twice daily for 5 days and repeated twice more at 5 day intervals. If unacceptable pancytopenia was evident, further therapy was delayed to permit neutrophil recovery to $0.5 \times 10^9 l^{-1}$.

Patients who did not achieve CR at this stage were consider to have failed the protocol.

After achieving CR, all patients, except for those who had received three 5-day blocks, received one further course of low-dose Ara-C and 6TG (Figure 1). This was given in 3, 4 and 5 day blocks, allowing neutrophil recovery between each block. Patients below 45 years of age with an HLA matched sibling donor then received BMT. ABMT was offered to the remaining medically fit patients during the last 3 years of the study.

All patients received prednisolone eye drops every 2 h for the first 10 days to reduce the incidence of conjunctivitis, prophylactic anti-emetics, and loperamide or pethidine to control diarrhoea. Granulocytopenic patients were nursed in single rooms.

Conditioning for BMT and ABMT was melphalan 110 mg m$^{-2}$ followed by single dose total body irradiation (TBI) to a midplane dose of 10 Gy (3 cGy min$^{-1}$) (Powles *et al.*, 1980*b*). BMT patients were nursed in protected environments. Oral prophylactic antibiotics included neomycin, colistin, amphotericin and nystatin. Patients developing fevers were treated empirically with intravenous broad spectrum antibiotic combinations. Fungal infections, documented or suspected, were treated with amphotericin. BMT patients received cyclosporin as prophylaxis against graft *versus* host disease (GVHD) (Powles *et al.*, 1980*b*). Those developing GVHD received high-dose methyl prednisolone (Kendra *et al.*, 1981). Marrow was not purged prior to ABMT, and patients received no subsequent maintenance chemotherapy.

### Results

A total of 30 cases (58%) achieved CR with the first course of treatment. A further five patients achieved CR (67%) with further courses of 5-day Ara-C and 6TG, without use of an anthracycline (Table I). Ten more (19%) with more than 10% blasts in their marrows after the first course of Ara-C and etoposide, obtained CR after entering the treatment arm containing adriamycin and daunomycin, giving an overall CR rate of 86.5% (45/52). Following the first course of treatment, the median interval to neutrophil recovery ($>0.5 \times 10^9 l^{-1}$) was 21 days (range 12 to >100 days), and to platelet recovery ($>100 \times 10^9 l^{-1}$) was 21 days (range 14 to >100 days). Median duration of hospitalisation was 27 days (range 19–115 days). It was longer for patients requiring the anthracycline arm of the protocol (Table I). Supportive measures during remission induction included a median of 11 units of blood, 45 units of platelets, 0.5 units of WBC and 20 days of i.v. antibiotics (Table II).

Of 25 patients with a monocytic component to their disease (FAB subtypes M4 and M5), 23 (92%) achieved CR, 17 (68%) without and six (24%) with an anthracycline. Seven failed to achieve CR and thus failed the protocol (Table I). Remission rates for patients receiving etoposide once daily were identical to those receiving it twice daily (Table III). The toxicity of Ara-C and etoposide is shown in Table IV.

**Table II** Supportive care during remission induction with BF11

| | *Median no.* | *Range* |
|---|---|---|
| Days in hospital | 27 | (19–115) |
| Units of blood | 11 | (3–33) |
| Units of platelets | 45 | (9–144) |
| Units of WBC | 0.5 | (0–15) |
| Days on i.v. antibiotics | 20 | (7–73) |

**Table III** Results of CR-1 and etoposide schedule

| | *Etoposide* | |
|---|---|---|
| | *Once daily (n = 23)* | *Twice daily (n = 29)* |
| Achieved CR-1 | 20 (87%) | 26 (89%)* |
| Continued CR-1 | 12 (52%) | 7 (24%)* |
| Relapsed | 5 (21%) | 13 (45%)* |
| after chemotherapy alone | 4 (17%) | 9 (31%)* |
| after ABMT | 1 (4%) | 4 (14%)* |
| after BMT | – | – |

*Not statistically significant.

**Table IV** Toxicity of high dose Ara-C and etoposide

| | | *Etoposide* | |
|---|---|---|---|
| | *Total (n = 52)* | *Once daily (n = 23)* | *Twice daily (n = 29)* |
| Diarrhoea | 41 | 20 | 21 |
| Abdominal pain | 24 | 10 | 14 |
| Vomiting | 16 | 5 | 11 |
| Intestinal obstruction | 3 | 1 | 2 |
| Meleana | 2 | 1 | 1 |
| Intestinal perforation | 1 | – | 1 |
| Skin rash | 11 | 6 | 5 |
| Conjuctivitis | 8 | 3 | 5 |
| Fits/drowsiness | 3 | – | 3 |
| Treatment related mortality | 5 | 3 | 2 |

Difference not statistically significant for any variable.

Major problems were gut-related. There was no statistical difference, however, in toxicity between groups receiving etoposide one or twice daily. Treatment-related mortality was 9.4% (5/52).

Of the 45 remitters, 20 had no further remission induction treatment, 14 received ABMT and 11 BMT while in CR 1 (Table V). Nineteen (42%) of these 45 patients continue in first CR (7/11 with BMT; 6/14 with ABMT and 6/20 with no treatment). Of the remaining 26, 17 relapsed (12/20 with no treatment and 5/14 after ABMT), and nine died while in remission (4/11 with BMT, 3/14 with ABMT and 2/20 with no treatment). Five of the 17 who relapsed did so within 3 months of achieving CR. There was only one CNS relapse (FAB subtype M5) in the entire group.

With a minimum follow-up of 90 days, the actuarial median duration of disease-free survival in CR1 was 321 days (range 13–911 days). Median duration of remission was shortest for the 29 patients who did not receive ABMT or BMT (180 days; range 13–782 days). This includes two patients who died of fungal infection within the first month, while still in CR1 (Table IV). Median duration of remission was 498 days (range 181–911) for ABMT and has not yet been achieved for BMT (range 118–906 days). The median interval from achieving CR to transplantation was 92 days for both autografts and allografts.

Quality of CR in patients receiving etoposide once daily is compared to the twice daily group in Table IV. Of the patients continuing in CR1, 12/23 belonged to the former and 7/29 to the latter group. Eleven of 17 (64%) who relapsed achieved second CR (eight with chemotherapy, two with BMT in relapse and one with ABMT in relapse). Six of these continue in second CR. Two of the four patients achieving CR2 with chemotherapy alone have subsequently received an autograft using second remission marrow. Outcome of the 5/11 patients not continuing in CR2 includes one in CR3 (after a second autograft in second relapse), one alive in second relapse and three dead (two in second relapse and one in CR2).

Currently, 26 (50%) of patients are in remission, 25 (48%)

**Table V** Status of first CR

| | *Chemotherapy alone (n = 20)* | *Allogenic BMT (n = 11)* | *Autologous BMT (n = 14)* | *Total (n = 45)* |
|---|---|---|---|---|
| Actuarial disease-free survival in CR-1 | | | | |
| median | 180 | [a] | 498 | 321 |
| range | 13–782 | 118–906 | 181–911 | 13–911 |
| Interval from CR to BMT | | | | |
| median | – | 92 | 92 | 92 |
| range | – | 45–157 | 6–342 | 6–342 |
| Current status | | | | |
| CCR | 6 | 7 | 6 | 19 |
| Died in CR | 2 | 4 | 3 | 9 |
| Relapsed | | | | |
| died with disease | 4 | – | 2 | 6 |
| achieved CR-2 | 8[b] | – | 3 | 11 |

[a]Not yet achieved. [b]CR-2 achieved with chemotherapy in five, BMT in two and ABMT in one of these patients.

are dead and one (2%) is alive with disease. Of the 25 who died, 11 had active disease, nine died of transplant-related causes (five infection, three GVHD, one progressive quadraparesis) and five had induction protocol toxicity (three infections, two gut toxicity).

A multivariant regression analysis was undertaken, in which age at presentation, sex, WBC count at diagnosis, blast percentage at presentation, FAB subtype and time to platelet and polymorph recovery after chemotherapy were considered as dependent variables. None showed any statistical significance with respect to achieving or remaining in CR.

### Discussion

Management of AML remains problematic because of failure to improve cure rates (Hurd, 1987; Powles *et al.*, 1980*a*; Mayer, 1987; Preisler *et al.*, 1987*a*; Cassileth *et al.*, 1987; Rohatiner *et al.*, 1988). This is partly attributable to lack of matched sibling donors for many patients, increased drug toxicity in the elderly, and a high incidence of relapse particularly in FAB subtypes with a monocytic component (Hurd, 1987; Mayer *et al.*, 1987; Peterson *et al.*, 1987).

Standard protocols containing Ara-C and daunorubicin with or without 6 thioguanine have recently been reported to give CR rates of 57–72% in *de novo* AML (Hurd, 1987; Preisler *et al.*, 1987*a*; Cassileth *et al.*, 1987; Capizzi *et al.*, 1987; Reiffers *et al.*, 1989). We obtained a CR rate of 67% without use of an anthracycline, and an overall CR rate of 86.5% where an anthracycline was used in ten (19%) patients. Remission was obtained in a single admission. Median duration of hospitalisation was shorter than for patients receiving the 3 plus 10 DAT regimen in the MRC 9 trial (Rees, 1989), and contrasts with previous reports which failed to demonstrate any benefit from addition of other chemotherapeutic agents to high-dose Ara-C alone for newly diagnosed AML (Gale *et al.*, 1987).

Low-dose Ara-C and 6TG maintained patients in remission during evaluation for BMT. As expected, median duration of first CR was shortest in patients receiving no further treatment after obtaining CR (Reiffers *et al.*, 1989). Of the 11 patients in first CR receiving allogeneic BMT, seven continue to be event-free survivors with a probability of surviving in continued CR of 62.3% at 18 months. No patient receiving BMT in CR1 has relapsed. There have been no adverse events after 10 months, and five patients are well up to 36 months from allograft.

Of the 19 patients who continue in first CR, 15 (80%) did not require an anthracycline. The proportion of patients continuing in first CR without an anthracycline (15/35; 43%) parallels that which did require an anthracycline (4/10; 40%). This would imply that efficiency of Ara-C and etoposide in inducing long-term DFS may be comparable to that achieved using an anthracycline-containing regimen (Mayer, 1987; Preisler *et al.*, 1987*b*; Cassileth *et al.*, 1987; Capizzi *et al.*, 1987). Patients receiving etoposide once daily fared better (52% CCR) than those receiving it twice daily (24% CCR) even though the initial CR rate was comparable (87% and 98%) respectively. Twice daily administration failed to prolong CR and caused an unacceptably high incidence of gut toxicity.

There was no evidence that any specific subgroup of AML fared worse with respect to disease characteristics at presentation. Specifically, patients with a monocytic component to their disease (FAB subtypes M4 and M5) did as well as others. Only one patient sustained a CNS relapse. Although not statistically significant, there is some indication that patients with M3 morphology fare better with inclusion of an anthracycline (Table I).

Treatment-related mortality during induction was less than 10% (5/52), and includes three infective deaths and two from gut toxicity. This is comparable to other protocols using high-dose Ara-C (Capizzi *et al.*, 1987; Gale *et al.*, 1987). No patient receiving etoposide once daily developed CNS toxicity. In general, toxicity was considerably less than previously reported in patients with relapsed and refractory acute leukaemias (Gore *et al.*, 1989). In the group as a whole, the majority of deaths were due to disease (Morra *et al.*, 1984), seven failing to remit and the remainder relapsing. Twice daily administration of etoposide failed to produce an improved anti-leukaemia effect.

We conclude that this protocol constitutes a safe, fast and effective induction regimen for *de novo* AML. Patients remitting after the first course of chemotherapy were discharged from hospital as rapidly as after 21 days, consolidation therapy being administered on an outpatient basis. Subsequent bone marrow transplantation was not compromised, results being comparable to those published using alternative induction regimens such as DAT (Helenglass *et al.*, 1987). Results for the group who received BMT in CR1 are comparable to those seen in the MRC AML 9 trial (personal communication, J.H. Rees).

Patients require long-term evaluation, but there is sufficient evidence to warrant further studies designed to ascertain optimal dosages to maximise chances of cure.

With recent emphasis on medical audit, it can be said that BF11 patients are in hospital for a shorter time than those treated in the MRC AML 9 DAT trial, and that they require less support and experience superior remission induction rates with a lower relapse probability.

Cost of BMT has not been considered here and requires evaluation, although BMT procedures are generally applicable in acute myeloid leukaemia, regardless of the chemotherapy induction regimen used to effect remission.

This study was funded by the Bud Flanagan Leukaemia Fund, the Leukaemia Research Fund and the Cancer Research Campaign, whose help is gratefully acknowledged. We are also grateful to Upjohn for providing the Ara-C.

### References

CAPIZZI, R.L., POWELL, B.L., COOPER, M.R. & 4 others (1987). Sequential high dose ara-C and asparaginase (HIDACAONase) induction therapy in 92 patients (pts) with newly diagnosed AML. *Proc. Am. Soc. Clin. Oncol.*, **6**, 164.

CASSILETH, P.A., BEGG, C.B., SILBER, R. & 9 others (1987). Prolonged unmaintained remission after intensive consolidation therapy in adult acute non lymphocytic leukaemia. *Cancer Treat. Rep.*, **71**, 137.

CHAMPLIN, R., HO, W., WINSTON, D. & 5 others (1987). Treatment of adults with acute myeloid leukaemia: prospective evaluation of high dose cytosine in combination chemotherapy and with bone marrow transplantation. *Semin. Oncol.*, **14**, Suppl. 1, 1.

GALE, R.P. & FOON, K.A. (1987). Therapy of acute myelogenous leukaemia. *Semin. Haematol.*, **24**, 40.

GORE, M., POWLES, R., LAKHANI, A. & 9 others (1989). Treatment of relapsed and refractory acute leukaemia with high dose cytosine arabinoside and etoposide. *Cancer Chemother. Pharmacol.*, **23**, 373.

HELENGLASS, G., LAKHANI, A., POWLES, R. & 8 others (1987). Bone marrow transplantation – the Marsden Experience. *Haematol. Oncol.*, **5**, 245.

HURD, D.D. (1987). Allogeneic and autologous bone marrow transplantation for acute nonlymphoblastic leukaemia. *Semin. Oncol.*, **14**, 407.

KENDRA, J., BARRETT, A.J., LUCAS, C. & 7 others (1981). Response of graft versus host disease to high dose methylprednisolone. *Clin. Lab. Haematol.*, **3**, 19.

MAYER, R.J. (1987). Current chemotherapeutic treatment approaches to the management of previously untreated adults with the *de novo* acute myelogenous leukaemia. *Semin. Oncol.*, **14**, 384.

MORRA, R., LAZZARINO, M., ALESSANDRINO, E., INVERADI, D., CANEVARI, A. & BERNASCONI, C. (1984). VP16-213 and cytosine arabinoside combination chemotherapy for refractory acute lymphoblastic leukaemia in adults. *Eur. J. Cancer Clin. Oncol.*, **20**, 1471.

O'DWYER, P., LEYLAND-JONES, B., ALONSO, M., MARSONI, S. & WITTES, R. (1985). Drug therapy: etoposide (VP16-213). Current status of an active anticancer drug. *N. Engl. J. Med.*, **312**, 692.

PETERSON, B.A. & LEVINE, G. (1987). Uncommon subtypes of acute nonlymphocytic leukaemia: clinical features and management of FAB M5, M6, and M7. *Semin. Oncol.*, **14**, 425.

POWLES, R.L., CLINK, H.M., SPENCE, D. & 12 others (1980). Cyclosporine A to prevent graft versus host disease in man after allogeneic bone marrow transplantation. *Lancet*, **i**, 327.

POWLES, R.L., CLINK, H.M., BANDINI, G. & 11 others (1980). The place of bone marrow transplantation in acute myelogeneous leukaemia. *Lancet*, **i**, 1047.

PREISLER, H., DAVIS, R.B., KIRSHNER, J. & 10 others (1987). Comparison of three remission induction regimens and two post induction strategies for the treatment of acute nonlymphoblastic leukaemia: a cancer and leukaemia group B study. *Blood*, **69**, 1441.

PREISLER, H.D., RAZA, A., BARCOS, M. & 14 others (1987). High dose cytosine arabinoside as the initial treatment of poor risk patients with acute non-lymphocytic leukaemia: a leukaemia intergroup study. *J. Clin. Oncol.*, **5**, 75.

REES, J.K.H. (1989). Chemotherapy of acute myeloid leukaemia in UK: past, present and future. *Bone Marrow Transpl.*, **4**, 110.

REIFFERS, J., GRASPARD, M.H., MARANINICHI, D. & 10 others (1989). Comparison of allogeneic and autologous bone marrow transplantation and chemotherapy in patients with AML in 1st remission: a prospective controlled trial. *Br. J. Haematol.*, **72**, 57.

RIVERA, G., AVERY, T. & ROBERTS, D. (1975). Response of L1210 to combinations of cytosine arabinoside and VM26 or VP16-213. *Eur. J. Cancer*, **11**, 639.

ROHATINER, A.Z.S., GREGORY, W.M., BASSAN, R. & 11 others (1988). Short-term therapy for acute myelogenous leukaemia. *J. Clin. Oncol.*, **6**, 218.